# Consumer Response to Corporate Hypocrisy From the Perspective of Expectation Confirmation Theory

**DOI:** 10.3389/fpsyg.2020.580114

**Published:** 2020-11-16

**Authors:** Wang Zhigang, Zhang Lei, Liu Xintao

**Affiliations:** ^1^Economics and Management School of Wuhan Sports University, Wuhan, China; ^2^Postgraduate School of Wuhan Sports University, Wuhan, China

**Keywords:** corporate social responsibility, corporate hypocrisy, expectation, negative emotions, negative behaviors

## Abstract

Based on the concept of hypocrisy perception, this paper studies and discusses consumers’ response to corporate social responsibility (CSR) hypocrisy perception, discusses the formation of consumers’ hypocrisy perception from the perspective of consumers’ expectation of CSR, and originally reveals the psychological and behavioral mechanisms of the generation of negative emotions and their role in consumer response. The results are as follows: (1) consumers’ CSR expectations and CSR perceived performance have a significant impact on their perception of hypocrisy; (2) consumers’ perception of hypocrisy has a significant impact on their negative emotions; (3) consumers’ negative emotions can have a significant impact on their attitudes and negative behaviors. The research results show that consumers’ expectations of CSR activities can affect consumers’ attitudes and behaviors, among which consumers’ perceived CSR performance, perceived hypocrisy, and negative emotions play an important role. In the implementation of CSR activities, enterprises should avoid making consumers have excessive expectations and appease their negative emotions, so as to improve the implementation effect of CSR activities.

## Introduction

Corporate social responsibility (CSR) is a strategic corporate tool for influencing consumers because it can encourage consumers’ positive responses toward the firm, such as increased purchase intention (Luo and Bhattacharya, 2006). To achieve support from consumers, many corporations build their positive social image by conducting CSR activities, such as poverty alleviation, disaster relief, and help for vulnerable groups (Brouwer et al., 2013). However, not all CSR activities conducted by corporations can lead to positive consumer responses toward the firm because many CSR activities, if not conducted appropriately, can lead to negative consumer responses, such as boycotting of the firm and its products. In 2008, a strong earthquake occurred in Sichuan Province, China, which resulted in substantial damages to life and property in the local area. China Vanke Co., Ltd., a leading provider of urban and rural construction and living services in China, and a leading enterprise in the real estate industry in China, and a real estate leader with annual profits of several hundred million dollars, donated only $286,143 to the Wenchuan earthquake in 2008, far less than the donation of enterprises of the same size (Country Garden, another large real estate enterprise in China, donated $1,920,945), and even less than the individual donation of some celebrities. This donation did not encourage consumers’ support for the company, but rather led to them boycotting the firm’s products, because consumers believed that the donated amount was too small considering the size of Vanke. Numerous people were angered by this small donation amount, which led to them boycotting Vanke.^1^ Nongfu Spring, a Chinese mineral water producer, launched a public welfare campaign in which they promised to donate 1 cent from each bottle of water to the welfare of children in poor areas. Through the public welfare campaign, the firm donated $738,826 to the welfare of children in poor areas. However, in 2008 alone, Nongfu Spring sold between 1.5 billion and 2 billion bottles of drinking water, according to industry analysts. “Based on this sales figure, Nongfu Spring should inject at least $2,216,476 into the fund every year, instead of Y 738,826.” Consumers considered this donation amount to be considerably lower than the amount that should have been donated based on the company’s sales; this led to consumers boycotting the company. Some consumers stated that they purchased Nongfu water because of its public welfare campaign. Therefore, they felt cheated by the firm, which hurt their sentiments.^2^

Given that CSR may lead to negative responses from consumers, Wagner et al. (2009) proposed the concept of corporate hypocrisy. In his research, Wagner et al. (2009) defined corporate hypocrisy as the belief that a firm claims to be something that it is not. Since then, corporate hypocrisy has been extensively studied. The corporate hypocrisy will make consumers have negative attitudes toward corporate brand (Santos and Casais, 2019). And corporate hypocrisy and consumers’ skepticism significantly influences perceived CSR and corporate reputation (Arli et al., 2019). Studies have evaluated the antecedents and consequences of corporate hypocrisy (Wagner et al., 2020). Several determinants can lead to corporate hypocrisy, such as CSR information (Scheidler et al., 2019) and authenticity perception (Guèvremont and Grohmann, 2018). Corporate donations are of value (Muhammad et al., 2019). In fact, a clear definition of corporate giving is a daunting task (Yuan et al., 2019). Corporate hypocrisy can have negative impacts on CSR beliefs, consumers’ attitudes toward the organization, corporate reputation (Wagner et al., 2009; Arli et al., 2017), and consumers’ purchase intentions (Guèvremont and Grohmann, 2018). Therefore, studies on corporate hypocrisy should further investigate the psychological process involved in the formation of the perception of corporate hypocrisy, such as the impact of attribution on hypocrisy (Wang and Zhu, 2020).

From the perspective of existing researches, researches on consumer CSR response have been very rich, and researches on consumer hypocrisy perception have also begun to attract attention. The current research on consumer hypocrisy perception response suggests that consumer hypocrisy perception can lead to negative attitudes and negative behaviors. But what is the mechanism? In addition, what is the current mechanism of consumers’ hypocritical perception? What factors contribute to the perception of consumer hypocrisy? These problems are blank in this field and need to be further discussed.

Consumers have a strong specificity in the perceptual response mechanism of corporate CSR hypocrisy, and their CSR expectations (Edell and Burke, 1987) and negative emotions (Antonetti and Maklan, 2016) have a strong impact. Stakeholders of an enterprise have their own expectations of CSR activities. If the CSR activities of an enterprise do not reach the level they expect, they will have a sense of hypocrisy (Edell and Burke, 1987). Consumers’ hypocritical perception of CSR activities will generate negative emotions (Antonetti and Maklan, 2016), and negative emotions will lead to consumers’ irrational behaviors (Ehsaneh and Shadi, 2013). According to this idea, this study introduces CSR expectations and CSR perceived performance factors to explain the formation of hypocritical perception. In addition, according to the consumption behavior in the attitude influence behavior theory, in this study, a negative attitude factor was added between consumer hypocrisy perception and consumer negative response to explain the formation mechanism between consumer hypocrisy perception and negative response.

An in-depth study of the psychological and behavioral mechanisms of consumer hypocrisy perception can help us to have a deeper understanding of how consumer hypocrisy perception triggers their negative response, so as to further deepen the research theory of consumer hypocrisy perception response.

In practice, consumers’ expectations regarding CSR is an important psychological factor influencing the formation of their perception of corporate hypocrisy. The negative emotion of consumers plays a crucial role in determining their hypocrisy perception, thereby leading to negative responses toward the firm. In the case of Vanke, the firm initially donated 2 million RMB to aid the reconstruction of the areas affected by the earthquake. However, this action caused consumers to boycott the firm’s products. In the same period, although many other small firms did not donate anything toward disaster relief, they did not encounter any negative responses from their consumers. Therefore, the question arises as to why a firm that made donations faced boycott from consumers, but companies that made no donations did not face any consumer dissatisfaction. The reason was the difference in the CSR expectations of consumers. Vanke is a large real estate corporation in China, and hence, consumers had high CSR expectations from the firm. Consumers expected the firm to donate a large sum of money toward disaster relief considering the huge profits it earned from the Chinese market. Therefore, consumers believed that it should take more social responsibility than other firms, which led to a perception of Vanke’s hypocrisy among consumers resulting in subsequent boycott of the firm’s products. However, consumers did not have the same CSR expectation from other small companies, and therefore, these companies did not encounter consumer dissatisfaction despite making no donations to disaster relief. In the case of Nongfu, although its donation of 5 million RMB to the welfare of children in poor areas was a large sum of money, it still did not meet the CSR expectation of consumers because, according to consumers, the company should have donated 15 million RMB based on their sales. Consumers were dissatisfied because the actual donation was lower than their expectation from the firm, which resulted in the formation of a perception of hypocrisy, negative emotions, and corresponding negative responses. Firms conducting CSR activities in the market are likely to face the impact of consumers’ CSR expectations based on their performance in these activities. Therefore, consumer expectations could be an independent factor influencing perceptions of hypocrisy regarding CSR. Moreover, the corresponding negative emotions of consumers play a crucial role in inducing negative consumer responses.

The current research on consumer hypocrisy perception response suggests that consumer hypocrisy perception can lead to negative attitudes and negative behaviors. However, the research gap is that the mechanism of the influence between consumer hypocrisy perception and negative reaction and from the perspective of expectation what factors affect consumer hypocrisy perception, which is not explored by existing research. The contribution of the study is to help us to understand the mechanism of influencing consumers’ negative response and the formation mechanism of consumers’ hypocrisy perception from the perspective of expectation.

The research method is first to explore the response mechanism of consumers’ hypocrisy perception through questionnaire survey, and adopts the research method designed by Wagner (Antonetti and Maklan, 2017). The relevant measurement scales of the questionnaire are based on the mature scales of relevant variables in the existing studies. Through the search of domestic and foreign literature, the existing scale of variables was collected, and then the discussion was conducted within the group. The measurement items were adjusted according to the research problems, and the final measurement scale was obtained. Then, questionnaires were distributed to consumers, data were collected, and research results were obtained through confirmatory factor analysis (CFA) and structural equation model test.

This study investigates the influence of consumers’ CSR expectations on their hypocrisy perception toward CSR and their corresponding responses. The role of negative emotions in inducing negative responses is also studied. According to expectation confirmation theory (ECT), the inconsistency between consumers’ expectation regarding CSR and their CSR performance perception can lead to the formation of hypocrisy perception toward CSR, which can result in negative consumer emotions and subsequently negative responses to the firm and its products. This study contributes to the understanding of the formation of and influence mechanism underlying corporate hypocrisy from a new perspective, thereby extending the theoretical and practical knowledge regarding corporate hypocrisy.

The main findings are as follows:

First, consumer perceptions of CSR hypocrisy are affected by their CSR expectation and perceptions of CSR performance.

Second, consumer perceptions of hypocrisy lead to negative emotions. The higher the consumer perception of CSR hypocrisy is, the higher their negative emotions are.

Third, consumers’ negative emotions can affect their attitudes and negative behaviors toward the firm. Consumers’ negative emotions can directly lead to their negative behaviors toward the firm.

The second part is the literature review and research hypothesis, and on this basis, the research model is established. The third part is the research method, including research design, variable measurement, and data collection. The fourth part is data analysis, including CFA and structural equation model test. The fifth part is the research conclusion and discussion. The discussion part includes three aspects: theoretical contribution, management inspiration, and research limitation.

## Literature Review

### Corporate Hypocrisy

Psychologically, a perception of hypocrisy is formed when there is a “distance between assertions and performance” (Shklar, 1984). This concept is also applicable to organizations because people perceive hypocrisy from various corporate activities (Aaker, 1997). Deceptive practices involving unsubstantiated claims, omission of information pertinent to a purchase, fraud, or other misdeeds, lead to perceptions of moral hypocrisy. However, deceptive practices, when discovered, may tarnish the company’s reputation (Swaen and Vanhamme, 2005) and lead to moral hypocrisy perception among consumers. Companies intend to build a moral or ethical image among consumers and other corporate audiences (Brown et al., 2006). In addition, the bad reputation of the company, information source type of CSR (Yoon et al., 2006), perceived motives of the CSR activities (Bae and Cameron, 2006), and inoculative communication strategies can lead to the emergence of a corporate hypocrisy perception among consumers. Corporate hypocrisy was defined by Wagner et al. (2009) as the belief that a firm claims to be something that it is not. This original definition was found to be insufficient because several facets of the concept remained undiscovered (Monin and Merritt, 2012; Laurent et al., 2014). Affective responses include negative emotional reactions such as anger, contempt, and disgust (Grappi et al., 2013); worsened attitudes (Brambilla et al., 2012); and reduced assessment of competence and skill of the firm (Stellar and Willer, 2018). Behavioral responses include avoidance of purchase from the firm (Haidt, 2003) through means such as reduced willingness to pay for a product (Guèvremont and Grohmann, 2018), boycotting of the firm (Braunsberger and Buckler, 2011), and spreading of negative word-of-mouth (Grappi et al., 2013).

Therefore, definitions of three aspects complementary to corporate hypocrisy were proposed, namely, moral hypocrisy, behavioral hypocrisy, and hypocrisy attributions. These definitions are clearer than the definition offered by Wagner et al. (2009) and provides a more accurate coverage of the theoretical properties of this construct (Wagner et al., 2020).

Cognitive responses include deliberate cognitive processing (Lee and Schumann, 2004) to resolve or reduce the incongruity (Ozanne et al., 1992), deliberate cognitive efforts (Kahneman, 2011), and trivialization of inconsistent information (Wagner et al., 2020).

Inconsistent practices such as a company’s divergent or incoherent statements, actions, policies, or procedures may cause consumers or other stakeholders to perceive the company as being unreliable, thereby leading to a perception of behavioral hypocrisy among consumers or other stakeholders (Wagner et al., 2020). The consequences of consumers’ hypocrisy perception include cognitive responses (Kahneman, 2011), affective responses (Haidt, 2003), and behavioral responses (Janney and Gove, 2011).

### Expectation Confirmation Theory

Oliver (1977) was the first to suggest that performance-specific expectation and expectancy disconfirmation play a major role in satisfaction decisions. Because individuals’ comparative judgment forms the input of their feeling of satisfaction (Watts, 1968; Weaver and Brickman, 1974), determining the effect of expectation and discrepancy perception on the satisfaction of consumers is crucial (Oliver, 1980). Consumers were thought to use expectations as a reference for making a comparative judgment (Oliver, 1980). Outcomes poorer than expected (a negative disconfirmation) are considered below this reference point, whereas those better than expected (a positive disconfirmation) are evaluated above this point (Oliver, 1980). Considerable evidence suggests that both expectation and disconfirmation affect postexposure product reactions (Swan, 1977; Linda and Oliver, 1979). Hence, confirmation or disconfirmation, affected by expectation, is crucial to satisfaction. According to ECT, consumers are likely to repurchase a product or service if the performance of the product meets their expectation (Oliver, 1980, 1993; Anderson and Sullivan, 1993). ECT explains the prepurchase expectation of a product or service and the confirmation or disconfirmation of this expectation based on the product or service performance, which can influence consumer satisfaction (Islam, 2014). ECT was applied to many product categories such as information systems in online banking (Bhattacherjee, 2001) and restaurants (Lee and Kim, 2020). Consumers first develop expectations regarding products or services, and then their real-life experience of using these products or services allows consumers to confirm or disconfirm their expectations (Lee and Kim, 2020). If the actual performance of the product or service exceeds the expectation, the expectation is confirmed. Otherwise, if the actual performance is inferior to the expectation, the expectation is disconfirmed (Churchill and Surprenant, 1982). The satisfaction and postpurchase behavior of consumers are based on the confirmation or disconfirmation of their expectations.

Studies have indicated that consumers’ CSR expectations can affect their perception of CSR (Lu and Powpaka, 2010). High expectations of CSR can result in positive feelings toward CSR if consumers cannot perceive the actual CSR performance (Wang and Wang, 2014). However, the role of consumers’ CSR expectations has not been discussed from the perspective of corporate hypocrisy. Therefore, the present study explores the influence of consumers’ CSR expectations on the formation of their hypocrisy perception toward CSR.

### Negative Emotions

Negative emotions are caused by moral transgressions. Ethical and social transgressions lead to negative emotional reactions in people, and violations of moral standards are associated with moral behaviors (Tangney et al., 2007). A study identified three types of negative emotions, namely contempt, anger, and disgust (Haidt, 2003). These negative emotions are often experienced together when an individual expresses clear disapproval of the actions of moral transgressors (Gutierrez and Giner-Sorolla, 2007).

Studies indicate that negative emotions occur as a consequence of assessments made by people, wherein they perceive that bad things that threaten their welfare have occurred. Some negative emotions, such as disgust and anger, are caused by situations that are perceived to be under the control of someone else (Lerner et al., 2004). People have negative emotions in response to a negative situation that another person could have controlled or avoid (Watson and Spence, 2007). In addition, the perceived fairness of a situation (Watson and Spence, 2007), the violation of human dignity (Grappi et al., 2013), and the violation of normative or moral standards can lead to negative emotions (Watson and Spence, 2007).

Negative emotions can influence evaluative judgments (Strack et al., 1985), risk assessment (Mayer et al., 1992) and persuasion (Rucker and Petty, 2004), negative word-of-mouth (Grappi et al., 2013), evaluation of events (Johnson and Tversky, 1983), negative implications (Petty et al., 1993), and complaint behaviors (Nyer, 1997). Negative messages that are framed when an individual is in a negative emotional state are more impactful (Wegener et al., 1995). According to mood repair theory, negative emotions drive people to seek situations more pleasant than those they are in, which results in consumers adapting coping strategies, such as confrontive coping driven by anger (Yi and Baumgartner, 2004).

The role of consumers’ negative emotions in CSR has been investigated only recently. Consumers’ negative emotions toward CSR are caused by the effect of ethical transgression (Grappi et al., 2013). If CSR activities do not satisfy consumers’ moral standards, consumers tend to exhibit negative emotions (Su et al., 2014). Irresponsible corporate behaviors tend to induce consumers’ negative emotions, resulting in unfavorable consumer behavior toward the firm (Xie et al., 2015). Consumers’ negative emotions also play an important role in their response to corporate hypocrisy regarding CSR (Wang and Zhu, 2020). The present study explored the occurrence of negative emotions in consumers and the role they play in consumers’ response to corporate hypocrisy.

### Conceptual Model

According to ECT, consumers’ expectation regarding a firm can influence their perception of the firm’s performance (Oliver, 1980); thus, their expectation of CSR can affect their perception of CSR performance. Moreover, consumers’ expectation and their perception of performance affect their psychological feelings (Oliver, 1977). Therefore, their CSR expectation and perception of CSR performance lead to their cognition toward CSR, including hypocrisy perception. Negative emotions can be induced in consumers if they feel cheated (Lerner et al., 2004). They form a perception of CSR hypocrisy when they believe that the firm is deceptive in its CSR activities. This perception of CSR hypocrisy can induce negative emotions in consumers. If consumers believe that a firm’s CSR activities are not adequately strong, they may be dissatisfied with the firm, thus forming negative emotions. If consumers have a perception of CSR hypocrisy, they form a bad impression of the firm. Consumers’ negative emotions lead to negative implications, which in turn lead to a decrease in attitude toward the firm (Petty et al., 1993). If consumers have a perception of CSR hypocrisy, they may conduct negative behaviors toward the firm in response to their perception of being cheated by the firm (Feldman and Russell, 1998). According to repair theory, consumers’ negative emotions toward the firm lead to negative behaviors as a coping strategy (Yi and Baumgartner, 2004). Consumers’ negative behaviors may also be explained by the theory of planned behavior, according to which consumers’ attitudes lead to their corresponding behaviors (Ajzen, 1991).

For CSR activities of enterprises, the higher the expectation level of consumers is, the greater their PERCEPTION of CSR performance will be, that is, the positive relationship between them. Consumers’ perception of hypocrisy is influenced by both their expectations and perceptions of CSR. The higher the expectation, the higher the perception of hypocrisy, and vice versa. Conversely, the higher CSR perceived performance, the less likely it is to generate hypocritical perception. Existing studies have proved that CSR activities of enterprises can have an impact on consumers’ mood. For CSR activities of enterprises, the higher consumers’ perception level is, the lower their negative emotion level will be. On the contrary, the lower the consumers’ perception level is, the higher their negative emotion level will be, that is, the two show an inverse relationship. In addition, consumers’ perception of hypocrisy can directly trigger consumers’ negative emotions. Consumers will have negative attitudes and negative behaviors toward enterprises. According to the theory of consumer behavior, consumer attitudes affect consumer behavior.

**FIGURE 1:**
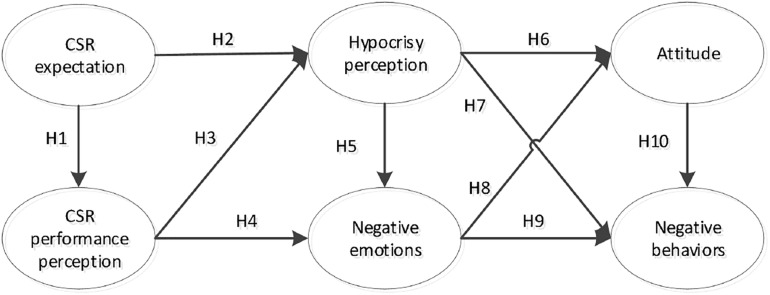
Conceptual model.

Based on the theoretical analysis, scholars arrived at the conceptual model (Figure 1).

## Hypotheses

An individual performs biased assimilation if the received information is not in accordance with their beliefs (Anderson and Kellam, 1992). Numerous studies have demonstrated that individuals evaluate the information they receive to support their own beliefs, and this process is known as biased assimilation (Greitemeyer and Schulz-Hardt, 2003). Expectation, like belief, can influence judgments even in the presence of unequivocal contrary evidence (Traut-Mattausch et al., 2004). Therefore, many studies have confirmed that individuals’ perceptions of price trends are affected by their initial expectations even if they received unequivocal evidence that their expectations were false (Greitemeyer et al., 2005). Expectation can influence perception in several ways; for example, it can bias perception under some conditions (Lange et al., 2018). Expectation shaped from previous experiences can influence individuals’ new perceptions (Sotiropoulos et al., 2011). Consumers’ expectations regarding CSR can influence their perception of CSR because of the assimilation effect. Therefore, we propose the following hypothesis.

H1: Consumers’ expectations of CSR positively influence their perceptions of CSR performance.

Consumers’ expectations of CSR are important because they tend to influence their perceptions of the firm (Poolthong and Mandhachitara, 2009). Consumers also expect the company’s CSR to exceed their expectation (Sen and Bhattacharya, 2001). According to expectancy violation theory (Burgoon et al., 1995), people perceive a company negatively if its behavior violates their social norms and expectations (Sohn and Lariscy, 2015). Consumers’ CSR expectation is an antecedent of their perception of moral inequity because their CSR expectation can determine their perception of corporate misconduct (Kim et al., 2019). Consumers have certain CSR expectations for a firm, the violation of which leads to negative consequences for the corporation because of the effect of moral transgressions (Grappi et al., 2013). To evaluate corporate behavior, consumers compare their CSR expectation to the conduct of the firm (Lindenmeier et al., 2012); any deviation from this expectation leads to consumer outrage, such as perceptions of the firm being unethical, unjust, or morally wrong (Krishna et al., 2018). Consumers’ perceptions of hypocrisy regarding CSR are affected by their CSR expectations. The higher consumers’ CSR expectations are, the greater their perception of hypocrisy regarding CSR will be. Therefore, we propose the following hypothesis.

H2: Consumers’ expectations of CSR positively influence their perceptions of hypocrisy regarding CSR.

Corporate hypocrisy occurs when a company’s actual CSR performance differs from its claims (Wagner et al., 2009), which means that the lower the perception of CSR performance is, the higher the perception of hypocrisy will be. An empirical study proved that consumers’ perceptions of CSR negatively influence their perception of hypocrisy regarding CSR (Kim et al., 2015). Consumers’ perceptions of CSR can influence their psychological perceptions of the firm, such as brand loyalty. Moreover, consumers’ perceptions of CSR can affect their evaluation of the firm’s CSR efforts (Salmones de los et al., 2005), consequently influencing their trust in the firm (Vlachos et al., 2009). In addition, an empirical study showed that customers’ CSR perception can influence their perception of the firm (Stanaland et al., 2011). Consumers’ perceptions of hypocrisy regarding the firm are an evaluation of the firm. Therefore, consumers’ perceptions of hypocrisy can also be affected by their perceptions of the firm’s CSR performance. Hence, we propose the following hypothesis.

H3: Consumers’ perceptions of CSR performance negatively influence their perceptions of hypocrisy regarding CSR.

Studies have indicated that CSR is an antecedent of customer emotions, which in turn affects their loyalty toward the firm. CSR activities can induce positive emotions in consumers, but they can also lead to negative emotional responses because of the effect of perceived corporate ethical transgression (Grappi et al., 2013). If the perceived performance of the firm does not meet consumers’ expectations, then consumers tend to have negative emotions toward the firm (Oliver, 1980). CSR can be perceived to facilitate important social life goals in terms of social justice, cooperation, and equality, which eventually leads to emotional responses (Romani et al., 2013). CSR initiatives can fulfill consumers’ moral interests of preventing harm to people; thus, if the consumers’ moral interest is not satisfied by CSR, negative emotions may be induced in them (Su et al., 2014). If a firm does not exhibit responsible behavior, consumers may form negative emotions, including disgust, anger, and contempt (Xie et al., 2015). Therefore, we propose the following hypothesis.

H4: Consumers’ perceptions of CSR performance negatively influence their negative emotions, including contempt, anger, and disgust.

The role of emotional reactions in consumers’ responses to corporate social irresponsibility (CSI) has been explored in marketing and consumer behavior studies (Kang et al., 2016; Antonetti and Maklan, 2017). Negative emotions lead to the arousal of and desire for behavior correction by punishing the perceived wrongdoer; therefore, negative emotions are often necessary to adequately react to immoral behavior (Fehr and Gächter, 2002). Studies have often used emotions to explain consumer behavior following a case of CSI (Antonetti, 2020), and negative emotions were considered mediators to explain why consumers attack irresponsible brands and engage in negative behaviors (Lindenmeier et al., 2012). Considerable research has shown that CSI can activate a wide and complex range of negative emotions, such as anger (Xie et al., 2015), contempt (Romani et al., 2013), moral outrage (Lindenmeier et al., 2012), fear (Antonetti and Maklan, 2016), sadness (Antonetti and Maklan, 2016), discontent (Romani et al., 2012), and disgust (Xie et al., 2015). The manifestation of intensive negative emotions is likely to explain revenge as consumers’ response to CSI and the company’s misbehavior (Antonetti, 2020). Although corporate hypocrisy might mediate the impact of CSI on consumer emotional reaction (Antonetti, 2020), just like CSI and corporate misbehavior, corporate hypocrisy can lead to consumers’ negative emotional responses (Wang and Zhu, 2020). Therefore, we propose the following hypothesis.

H5: Consumers’ perception of hypocrisy regarding CSR positively influences their negative emotions, including contempt, anger, and disgust.

Consumers’ attitudes toward the firm are affected by their perceptions of CSR initiatives (Karen et al., 2006) as well as their perceptions of corporate hypocrisy. Corporate hypocrisy can both directly and indirectly negatively affect consumers’ attitude toward the firm (Wagner et al., 2009), which was further validated by Shim and Kim (2015). Corporate hypocrisy has a destructive impact on a consumer’s attitudes toward the firm when an inconsistency between the firm’s promises and actions is observed (Crowther, 2004). An empirical study proved that corporate hypocrisy can negatively influence consumers’ attitude toward a firm through their CSR beliefs and perceived corporate reputation (Arli et al., 2017). Therefore, we propose the following hypothesis.

H6: Consumers’ perceptions of hypocrisy negatively influence their attitudes toward the firm.

Consumers’ perceptions of CSR can affect their behaviors (Peloza and Shang, 2011), such as purchase intention (Karen et al., 2006) and product recommendation (García-Jiménez et al., 2017). Environmentally irresponsible behavior by corporations can lead to negative consumer behaviors, including negative word-of-mouth, complaints, and boycotts (Xie et al., 2015). Similarly, consumers’ perceptions of corporate hypocrisy can lead to negative behaviors. The consequence of corporate hypocrisy could include serious boycotts from consumers (Xiao et al., 2013) and a refusal to buy products and services from such companies (Amélie, 2019). If consumers perceive CSR as hypocritical, they exhibit negative behavioral responses toward the firm to express their dissatisfaction. Therefore, we propose the following hypothesis.

H7: Consumers’ perceptions of hypocrisy positively influence their negative behaviors toward the firm, including negative word-of-mouth, complaints, and boycott.

The impact of emotions on attitude has been confirmed by many studies (López-Mosquera and Sánchez, 2010). Individuals’ emotions have direct impacts on their attitude formation (Edell and Burke, 1987). Nameghi and Shadi (2013) found that consumers’ emotions related to the environment were significantly associated with consumers’ attitude. The arousal of emotions in consumers can affect their attitudes (Yoo et al., 1998); emotional experiences can produce positive or negative beliefs, which in turn influence consumers’ attitudes (Fishbein and Ajzen, 1975). Moreover, negative or positive emotional experiences of consumers can produce a mood that affects their evaluation of the firm, which can affect their judgments related to the firm (Gardner, 1985). Individuals tend to express dislike toward or avoid intense negative emotional stimuli and prefer positive emotional stimuli (Moore and Harris, 1996). Hence, consumers’ negative emotions induced by corporate hypocrisy damage their attitude toward the firm. Therefore, we propose the following hypothesis.

H8: Consumers’ negative emotions negatively influence their attitudes toward the firm.

Emotions directly influence behavior because emotions can arouse and drive behaviors (Baumeister et al., 2007). Individuals’ emotional responses to an event can lead to corresponding behavioral responses (Collins, 1996). Therefore, the effect of emotion on consumer behavior has attracted considerable research attention (Holbrook and Hirschman, 1982). Consumer behaviors, such as impulsive and compulsive purchasing, are significantly influenced by emotions such as happiness and sadness (Hirschman and Stern, 1999). Many studies have shown that the positive and negative emotions that consumers associate with the consumption play key roles in determining their future behavioral intention (Oliver, 1993; Richins, 1997; Barsky and Nash, 2002). Positive emotions induced by the consumption are associated with positive future behavioral intention, and vice versa (Wong, 2004). Irresponsible corporate behavior induces negative behaviors toward the firm in consumers, such as negative word-of-mouth, complaints, and boycotts (Xie et al., 2015). In addition, consumers’ negative behaviors are generated through their negative emotions arising from perceptions of hypocrisy regarding CSR (Wang and Zhu, 2020). Therefore, we propose the following hypothesis.

H9: Consumers’ negative emotions positively influence their negative behaviors toward the firm, including negative word-of-mouth, complaints, and boycott.

Fishbein and Ajzen (1975) explained the relationship between individuals’ attitudes and their behavioral intentions by using the theory of reasoned action (Fishbein and Ajzen, 1975). Therefore, the concept of attitude has been considered important in understanding human behavior (Peter and Olson, 2010). Since Fishbein and Ajzen proposed this explanation, studies have increasingly evaluated the effect of consumers’ attitude on their behaviors in various contexts (Jung et al., 2014). Consumers’ attitude toward the firm can affect their behaviors, such as the contribution of knowledge to the user community (Chen et al., 2013) and interaction with other users. Consumers’ positive attitudes toward the firm can lead to favorable behaviors, whereas their negative attitudes toward the firm can lead to unfavorable behaviors. Hence, consumers’ attitudes, which are influenced by the environmental responsibility initiatives of firms, can influence their negative behaviors toward the firm (Xie et al., 2015). Therefore, we propose the following hypothesis.

H10: Consumers’ attitudes toward the firm negatively influence their negative behaviors toward the firm, including negative word-of-mouth, complaints, and boycotts.

Figure 1 presents the conceptual model of this study. Consumers’ CSR expectations can affect their perceptions of CSR performance, which in turn affects their perceptions of hypocrisy. Consumers’ perceptions of CSR performance and hypocrisy both influence the formation of their negative emotions. Consumers’ perceptions of hypocrisy can negatively influence their attitudes toward the firm and induce negative behaviors. Moreover, consumers’ negative emotions have a negative impact on their attitudes toward the firm, leading to their negative behaviors. Consumers’ attitudes toward the firm can also negatively affect their behaviors toward the firm. In the conceptual model, consumers’ CSR expectations are defined as their expected strength of CSR activities. Consumers’ CSR performance perception refers to the CSR strength perceived by consumers. Negative emotions include three dimensions: contempt, anger, and disgust. Negative behaviors include three dimensions, namely, negative word-of-mouth, complaint, and boycott, which are important outcome variables of consumers’ response.

## Research Method

### Research Design

The research scope of the study is about exploring the formation mechanism of consumers’ hypocrisy perception and the factors influencing the formation of consumers’ hypocrisy perception.

The research method is first to explore the response mechanism of consumers’ hypocrisy perception through questionnaire survey, and adopts the research method designed by Wagner (Antonetti and Maklan, 2017). The relevant measurement scales of the questionnaire are based on the mature scales of relevant variables in the existing studies. Through the search of domestic and foreign literature, the existing scale of variables was collected, and then the discussion was conducted within the group. The measurement items were adjusted according to the research problems, and the final measurement scale was obtained. Then, questionnaires were distributed to consumers, data were collected, and research results were obtained through CFA and structural equation model test.

During the process of survey, respondents first read a description of a simulated situation related to the hypocritical behavior of a firm. Then, they were asked to answer the questions that followed based on their actual feelings. The questions in the questionnaire were related to consumers’ expectations of corporate CSR behavior, perception of corporate CSR performance, perception of corporate hypocrisy, negative emotions, and attitudes and negative behaviors toward the company.

### Measurement

The measurement scales used in this study were compiled from the maturity scales used in previously published studies. The research team first collected relevant scales from the literature, adjusted the items according to the research context after a discussion with the team members, and then determined the final scales. The study measured six variables: CSR expectation, CSR performance perception, hypocrisy perception, negative emotions, attitude, and negative behaviors. Negative emotions and negative behaviors were second-order variables. These variables were scored on a seven-point Likert-type scale, with a score of 1 denoting *strongly disagree* and 7 denoting *strongly agree*.

Corporate social responsibility expectation and CSR performance perception were measured according to the scale used by Ludong (Lu and Powpaka, 2010), which includes four items. Hypocrisy perception was measured using the scale used by Wagner et al. (2009), which includes three items. Attitude was measured using the scale used by Wagner et al. (2009), which includes four items. The scale provided by Xie et al. (2015) was used to measure three dimensions of negative emotions: contempt, anger, and disgust. Each emotion was assessed using three items. Negative behaviors were also measured according to the scale by Xie et al. (2015) in terms of three dimensions: negative word-of-mouth, complaints, and boycotts. These three domains comprised three, four, and two items, respectively. The specific items of the scales are listed in Table 2.

### Data Collection

The questionnaire data were collected by sending questionnaires to consumers. The survey was conducted using a face-to-face method to distribute and collect the questionnaires. A total of 350 questionnaires were distributed and 326 questionnaires were collected, of which 302 were valid questionnaires, resulting in a recovery rate of 93.1% and an effectiveness rate of 92.6% (incomplete questionnaires were eliminated).

During the survey, the gender and age of the recipients as well as the timing of distribution between working days and weekends and working hours and rest hours were recorded to ensure the representativeness of the sample. The composition of the sample of surveyed consumers is presented in Table 1.

**TABLE 1:** Recipient characteristics.

Classification Indicator	Percentage (%)	Classification Indicator	Percentage (%)
**Gender**		**Age**	
Male	50.2	12–24	45.0
Female	49.8	25–39	39.1
**Marital status**		40–55	13.9
Married	39.6	>55	2.0
Single	60.4	**Monthly income** ($)	
**Religious**		<74	2.6
Yes	11.2	74–147	9.3
No	88.8	148–295	25.5
**Education**		296–443	15.9
Elementary school or below	1.0	444–739	20.2
Junior high school	6.0	740–1181	14.9
Senior high school	12.3	1182–1478	6.3
Junior college or undergraduate	67.9	>1478	5.3
Postgraduate or higher	12.8		

## Exploratory Factor Analysis

After estimating the reliability of scales by Cronbach’s alpha, all 33 items are employed in the exploratory factor alpha, all 33 items are employed in the exploratory factor analysis (EFA). The results of testing the validity of measures (variables) by the exploratory factor analysis show that KMO = 0.951, Sig. (Bartlett’s test) = 0.000 < 0.005, Initial Eigenvalues = 79.829 > 50%. Thus, all scales are appropriate for CFA at the next part. The final results of the exploratory factor analysis are illustrated in Table 2.

**TABLE 2:** Exploratory factor analysis (EFA).

Items		Components					
		1	2	3	4	5	6
CSRE1					0.828		
CSRE2					0.847		
CSRE3					0.842		
CSRE4					0.823		
CSRPP1						0.795	
CSRPP2						0.777	
CSRPP3						0.806	
CSRPP4						0.768	
HP1							0.688
HP2							0.707
HP3							0.754
Negative emotions	Contempt1		0.769				
	Contempt2		0.746				
	Contempt3		0.732				
	Anger1		0.739				
	Anger2		0.746				
	Anger3		0.723				
	Disgust1		0.748				
	Disgust2		0.754				
	Disgust3		0.726				
Attitude1				0.885			
Attitude2				0.904			
Attitude3				0.874			
Attitude4				0.808			
Negative behaviors	NW1	0.680					
	NW2	0.786					
	NW3	0.805					
	Complaint1	0.853					
	Complaint2	0.871					
	Complaint3	0.880					
	Complaint4	0.867					
	Boycott1	0.813					
	Boycott2	0.765					
Kaiser–Meyer–Olkin (KMO) measure of sampling adequacy				0.951			
Sig. of Bartlett’s test of sphericity				0.000			
Cumulative%				79.829			

## Data Analysis

### Confirmatory Factor Analysis Measurement Model

Descriptive statistics were performed using SPSS16.0 to identify missing, outliers, and also to check the assumption of normality. SPSS16.0 and AMOS 18.0 were used for CFA and structural equation modeling (SEM). For the goodness-of-fit measures, we used chi square test (χ^2^), standardized root-mean-square residual (SRMR), goodness-of-fit index (GFI), adjusted GFI (AGFI), Parsimony GFI (PGFI), Tacker–Lewis index (TLI), comparative fit index (CFI), root-mean-square error of approximation (RMSEA; Raykov et al., 1991). The acceptance criteria of these GFIs are shown in Table 3.

**TABLE 3:** Questionnaire items.

Latent variable		Item	Load (>0.6)	Standard deviation	AVE (>0.5)	Composite reliability (>0.6)	Cronbach’s α
CSR Expectation		I expect the company to actively help the vulnerable social groups.	0.836	0.123	0.720	0.911	0.912
		I expect the company to promote regional social development by supporting the construction of public facilities.	0.872				
		I expect the company to surpass the goal of only creating profits and take responsibility as a member of society.	0.835				
		I expect the company to actively conduct activities that contribute to the whole society.	0.851				
CSR Performance Perception		I think the company is actively helping the vulnerable social groups.	0.809	0.198	0.678	0.894	0.895
		I think the company really promotes the development of regional society by supporting the construction of public facilities.	0.797				
		I think the company has gone beyond the goal of only creating profits and fulfilled its responsibility as a member of society.	0.842				
		I think the company has actively conducted activities that contribute to the society.	0.844				
Hypocrisy Perception		The company does not want to assume social responsibility.	0.828	0.154	0.760	0.905	0.903
		The actual social responsibility behaviors of the company are not as good as those advertised.	0.898				
		Taking social responsibility is merely symbolic for the company, and the actual intention is to achieve other purposes.	0.888				
Negative Emotions	Contempt	I scorn the behavior of the company.	0.900	0.205	0.863	0.950	0.950
		I disdain the behavior of the company.	0.941				
		I despise the behavior of the company.	0.945				
	Anger	I am unhappy with the behavior of the company.	0.951	0.224	0.908	0.968	0.967
		I feel indignant toward the behavior of the company.	0.958				
		I am furious with the behavior of the company.	0.949				
	Disgust	I am disgusted with the behavior of the company.	0.946	0.234	0.882	0.957	0.957
		I dislike the behavior of the company.	0.966				
		I detest the behavior of the company.	0.905				
Attitude		In general, I like the company.	0.901	0.186	0.779	0.933	0.930
		In general, I think the company is good.	0.933				
		In general, I think the company is pleasant.	0.938				
		In general, I have a favorable attitude toward the company.	0.744				
Negative Behaviors	Negative Word -of -Mouth	I will tell my relatives, friends, and others that the company is not good.	0.885	0.197	0.821	0.932	0.933
		I will advise my relatives, friends, and others not to apply to the company.	0.928				
		I will tell my relatives, friends, and others that the company has done a lot of bad things.	0.905				
	Complaints	I will complain directly to the company.	0.910	0.197	0.853	0.959	0.958
		I will complain to the media.	0.925				
		I will complain to the government or industry authorities.	0.938				
		I will complain to the people in the company.	0.920				
	Boycott	I will tell other companies not to conduct business with this company.	0.921	0.219	0.843	0.915	0.914
		I will tell my friends not to buy products from the company.	0.915				

The reliability and validity of the data were verified through CFA by using AMOS 16.0 software. The following results indicated that the measurement model demonstrated acceptable goodness of fit: χ^2^(302) = 728.431, χ^2^/df = 1.667, root-mean-square residual = 0.077, GFI = 0.874, AGFI = 0.838, TLI = 0.970, CFI = 0.975, and RMSEA = 0.047. As illustrated in Tables 3, 4, the composite reliability (CR) of variables exceeded the recommended level of 0.6, and the average variance extracted (AVE) was also higher than the recommended level of 0.50, thus indicating that the measurement of variables was highly reliable. The normalized factor loading of structural variables was higher than 0.6 and was significant at α = 0.01, which indicates that the scale has a high degree of convergent validity. Moreover, the square root of all AVEs was greater than the correlation coefficient of the corresponding rows and columns, which indicates that the scale has a high discriminant validity.

**TABLE 4:** Latent variable relationships.

	CSR Expectation				CSR Performance Perception				CSR Hypocrisy Perception			Negative Emotion									Attitude				Behavior								
												Contempt			Anger			Disgust							Negative Word-of-Mouth			Complaint				Boycott	
	A1	A2	A3	A4	B1	B2	B3	B4	E1	E2	E3	F1	F2	F3	G1	G2	G3	H1	H2	H3	I1	I2	I1	I4	J1	J2	J3	K1	K2	K3	K4	L1	L2
**Descriptive statistics**																																	
Average	6.05	5.85	5.92	5.95	5.19	5.12	4.96	5.17	3.97	4.34	4.18	3.50	3.39	3.36	3.40	3.30	3.32	3.43	3.39	3.38	4.25	4.37	4.36	4.56	3.32	3.31	3.34	3.15	3.12	3.14	3.15	3.14	3.28
Standard deviation	1.24	1.17	1.28	1.27	1.58	1.51	1.57	1.51	1.39	1.40	1.46	1.60	1.58	1.62	1.66	1.61	1.61	170	1.70	1.66	1.52	1.52	1.59	1.52	1.57	1.63	1.60	1.56	1.61	1.62	1.60	1.64	1.74
Correlation coefficient																																	
CSR expectation	(0.849)																																
CSR performance perception	0.545				(0.823)																												
CSR hypocrisy perception	0.289				−0.005				(0.871)																								
Contempt	0.027				−0.105				0.697			(0.943)																					
Anger	0.040				−0.097				0.662			0.932			(0.962)																		
Disgust	0.060				−0.125				0.685			0.930			0.951			(0.939)															
Attitude	0.336				0.557				−0.014			−0.120			−0.128			−0.128			(0.883)												
Negative word -of -mouth	0.093				−0.035				0.637			0.807			0.811			0.811			−0.005				(0.916)								
Complaint	0.013				−0.074				0.542			0.772			0.781			0.754			−0.061				0.884			(0.924)					
Boycott	0.049				−0.081				0.601			0.830			0.842			0.842			−0.884				0.911			0913				(0.918)	

*Diagonal data are the square roots of the AVEs.*

Two second-order variables (negative emotions and negative behaviors) were also analyzed through CFA. The results of the first-order and second-order CFAs show that the factor load, CR, AVE, fit index of the model, and other indicators of each test item meet the relevant standards. This result indicates that the measurement model of the second-order variable has good reliability and validity.

### Test of the Structural Model

**FIGURE 2:**
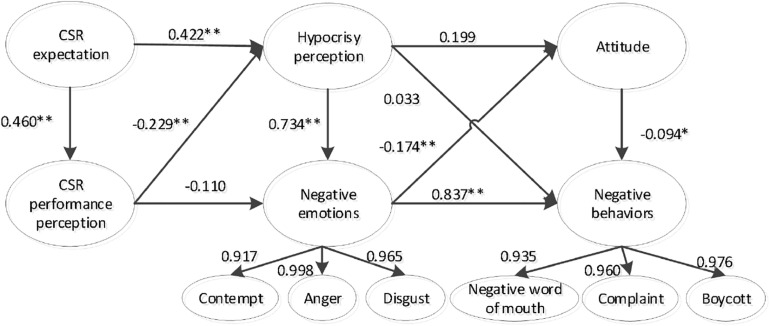
Path analysis of the structural equation model. Note. *p < 0.05, **p < 0.01.

We used structural equation modeling to explore the consumers’ response mechanisms to hypocrisy perception (Figure 1). The results are illustrated in Figure 2. The following statistics indicated that structural model fit the data well: χ^2^(302) = 788.388, χ^2^/df = 1.706, GFI = 0.864, AGFI = 0.835, TLI = 0.968, CFI = 0.972, and RMSEA = 0.048.

The standardized path coefficients of the structural model are displayed in Figure 2. The results revealed that consumers’ CSR expectations had a significant effect on their perceptions of hypocrisy (γ = 0.422, *p* < 0.01) and CSR performance (γ = 0.460, *p* < 0.01). Consumers’ perceptions of hypocrisy had a significant effect on their negative emotions (γ = 0.734, *p* < 0.01), but no significant effect on their attitudes toward the firm (γ = 0.199, *p* > 0.05) or negative behaviors toward the firm (γ = 0.033, *p* > 0.05). Consumers’ perceptions of CSR performance had a significant effect on their perceptions of hypocrisy (γ = −0.229, *p* < 0.01), but no significant effect on their negative emotions (γ = −0.110, *p* > 0.05). The negative emotions of consumers had a significant effect on their attitudes (γ = −0.174, *p* < 0.05) and negative behaviors toward the firm (γ = 0.837, *p* < 0.01). Consumers’ attitudes toward the firm had a significant effect on their negative behaviors toward the firm (γ = −0.094, *p* < 0.05). These results supported H1, H2, H3, H5, H8, H9, and H10, but H4, H6, and H7 were not supported.

## Research Results

Consumers’ expectation of CSR activities has a positive impact on their perceived CSR performance. For CSR activities of enterprises, the higher their expectation level is, the greater their perceived CSR performance will be. Due to the role of expectancy Assimilation (Assimilation), the level of human perception would increase with the improvement of expectation level.

Consumers’ expectations of CSR activities positively affect their perception of hypocrisy. Consumers’ perception of CSR activities negatively affects their perception of hypocrisy. Human hypocrisy perception is the result of comparison, and consumers’ hypocrisy perception will be jointly influenced by their expectation and perception of CSR. If consumers’ perceived CSR performance is lower than their expected CSR level, they will think that CSR activities of the enterprise are just “cosmetic,” instead of really assuming their due social responsibility. Then they will have hypocritical perception, and the greater the gap, the higher the level of hypocritical perception. The higher the expectation of consumers for CSR activities, the more likely they are to be hypocritical.

Consumers’ perceived CSR performance negatively affects their negative emotions. In general, CSR activities of enterprises can strengthen the positive emotions of consumers and weaken the negative emotions of consumers. For CSR activities of enterprises, the higher consumers’ perception level is, the lower their negative emotion level will be. On the contrary, for CSR activities of enterprises, the lower the consumers’ perception level is, the higher their negative emotion level will be.

Consumers’ perception of corporate hypocrisy can positively affect their negative emotions. The company’s irresponsible behavior is a kind of moral transgression, which makes consumers feel that the company is morally wrong. Existing research suggests that consumers will experience negative emotions, including contempt, anger and disgust, if a company engages in irresponsible behavior toward the environment.

Consumers’ perception of hypocrisy negatively affects their attitude toward business. When consumers find that enterprises have immoral behaviors including hypocrisy, they will take punitive actions against enterprises, such as boycotting their products.

Consumers’ perception of hypocrisy will positively influence their negative behavior toward enterprises. For CSR activities of enterprises, if consumers have hypocritical perception, they will think that enterprises do not really want to take social responsibility, which will have a negative impact on the attitude of enterprises and take behaviors that are not conducive to enterprises.

Negative emotions will negatively affect consumers’ attitude toward enterprises, and the higher the level of negative emotions, the worse their attitude toward enterprises will be. In the process of interaction between consumers and enterprises, if consumers have negative emotions, they will retaliate against enterprises in a series of ways, such as switching purchase.

Negative emotions will positively affect consumers’ negative behaviors toward enterprises. The negative emotions caused by the irresponsible behaviors of enterprises can lead to the negative behaviors of consumers toward enterprises, including negative reputation, complaints, and boycotts.

Consumers’ attitude toward business can negatively affect their negative behaviors. Consumers’ positive attitude will lead to their positive behavior toward the enterprise, while consumers’ negative attitude will lead to their negative behavior toward the enterprise. The better consumers’ attitude toward the business, the less negative behavior (negative word of mouth, complaints, and boycotts) they will have.

The worse consumers feel about the business, the more negative behavior (negative word of mouth, complaints, and boycotts) they have.

## Research Discussion and Conclusion

### Discussion

Corporate social responsibility activities conducted by firms can lead to favorable consumer attitude and behaviors toward the firm (Luo and Bhattacharya, 2006). Consumers’ responses to CSR include long-term loyalty (Marin et al., 2008), advocacy behavior (Du et al., 2007), purchase intention (Chomvilailuk and Butcher, 2010), and brand attitude (Howard and Barry, 1994). Studies on corporate hypocrisy have revealed that CSR can induce negative attitudes in consumers toward the firm (Wagner et al., 2009). This study revealed that CSR can influence consumers’ attitudes and behaviors toward the firm in the context of hypocrisy perception, which is consistent with previously published studies. However, in the context of hypocrisy perception, CSR can negatively influence consumers’ responses. This study confirmed that consumers can exhibit negative responses to the CSR activities of the firm in the context of hypocrisy perception, which differs from the findings of previous studies. Thus, in addition to positive responses to CSR, in the context of hypocrisy perception, consumers can exhibit negative responses to CSR, including negative attitudes and negative behaviors (i.e., negative word-of-mouth, complaints, and boycott). Therefore, this study proposes a new type of consumer response to CSR, namely, negative response, in the context of corporate hypocrisy.

Consumer responses to CSR include changes in attitude and behavior (Xie et al., 2019). Previous studies have rarely highlighted consumers’ emotional responses to CSR because consumers usually do not exhibit intense emotional responses to CSR in a normal context. However, in the context of hypocrisy perception, consumers exhibit strong emotional responses because they feel cheated by the firm. Consumers’ emotional responses to CSR are different in the context of hypocrisy perception, which has not been discussed in previous CSR studies. Therefore, apart from changes in attitude and behavior, consumers’ emotional response to CSR is a new type of consumer response to CSR explored in this study.

Previous studies have revealed that inconsistent information regarding CSR (Wagner et al., 2009), consumer attribution (Wang and Zhu, 2020), time between talk and action (Christensena et al., 2020), and corporate reputation (Shim and Yang, 2016) can influence the formation of consumers’ perception of hypocrisy regarding CSR. This study explored the impact of consumers’ expectations of CSR on the formation of their perception of hypocrisy regarding CSR from a new perspective; the results revealed that consumers’ CSR expectations and CSR performance perceptions can affect the formation of their hypocrisy perceptions regarding CSR. Therefore, this study further identified that consumers’ psychological perceptions can affect the formation of their hypocrisy perceptions.

### Research Conclusion

This study explored the psychological and behavioral mechanism underlying the formation of consumers’ perceptions of CSR hypocrisy and the impact on the firm. The role of consumers’ expectations of CSR and negative emotions are confirmed in the process. Consumer expectation of a firm’s CSR plays an important role in the formation of consumers’ perceptions of hypocrisy, whereas consumers’ negative emotions play a crucial role in the impact of consumers’ perceptions of hypocrisy on their attitudes and behavioral responses. The conclusions are as follows.

First, consumer perceptions of CSR hypocrisy are affected by their CSR expectation and perceptions of CSR performance. Consumer perceptions of hypocrisy are strengthened by high CSR expectations and low CSR performance perceptions. Some stimuli, such as corporate propaganda, corporate behaviors, corporate resources, and the severity of social problems, encourage consumers’ expectations regarding the firm’s CSR. Consumers compare their perception of CSR performance with their CSR expectation for the firm. A high CSR expectation and a low CSR performance perception lead to a high perception of hypocrisy regarding CSR among consumers.

Second, consumer perceptions of hypocrisy lead to negative emotions. The higher the consumer perception of CSR hypocrisy is, the higher their negative emotions are. If consumers perceive that firms conduct CSR activities not to solve social problems but merely to build a favorable image or to achieve other purposes through the implementation of CSR, then consumers will be dissatisfied with the corporate behavior. Thereafter, they will form negative emotions as a consequence of their perception of hypocrisy in the firm.

Third, consumers’ negative emotions can affect their attitudes and negative behaviors toward the firm. Consumers’ negative emotions can directly lead to their negative behaviors toward the firm. Consumers’ negative emotions can also affect their attitudes toward the firm, thus leading to their negative behaviors toward the firm. The higher the negative emotions of consumers are, the stronger their negative behaviors toward the firm are. Consumer perception of hypocrisy causes negative emotions, which in turn leads to their corresponding negative behaviors against the firm, including negative word-of-mouth, complaints to the government and media, and boycott of the products of the firm. The higher the level of consumers’ negative emotions caused by their perception of hypocrisy is, the more intensive the negative behaviors toward the firm are, which ultimately has a strong negative impact on the firm.

### Theoretical Contributions

This research revealed the influence mechanisms of CSR expectations, CSR performance perceptions, hypocrisy perceptions, negative emotions, negative attitudes, and negative behaviors of consumers in the context of consumers’ perceptions of hypocrisy regarding CSR. The theoretical contributions of this study are as follows.

First, this study discussed the psychological and behavioral response mechanisms underlying consumers’ perceptions of hypocrisy regarding CSR, which opens a “black box” of response mechanisms underlying consumers’ perceptions of hypocrisy. Studies have identified that consumers’ perceptions of hypocrisy affect their attitudes and behaviors (Wagner et al., 2009), but the mechanism underlying this influence has not been explored. Negative emotion is an important variable that plays a crucial role in consumers’ responses to their hypocrisy perception. Consumers’ perceptions of hypocrisy regarding CSR arouse negative emotions, which in turn induce negative behaviors toward the firm. This study revealed that consumers’ negative emotions are an intermediary between consumers’ perceptions of hypocrisy and their responses, which further strengthens the research on consumers’ responses to their perceptions of hypocrisy regarding CSR.

Second, based on ECT, this study discussed the formation of consumers’ perceptions of hypocrisy from the viewpoint of their CSR expectations, which enriches the research on the formation of consumers’ perceptions of hypocrisy regarding CSR. Studies have indicated that consumer attribution has a significant impact on the formation of perceptions of hypocrisy regarding CSR (Wang and Zhu, 2020). The present study further explored the role of consumers’ CSR expectations and CSR performance perceptions in the formation of their perceptions of hypocrisy regarding CSR. Both consumers’ CSR expectations and their CSR performance perceptions significantly influenced the formation of their perceptions of hypocrisy regarding CSR. This finding further improves the understanding of the formation of consumers’ perceptions of hypocrisy regarding CSR as well as the underlying formation mechanism.

Third, this study adopted negative behaviors as a type of consumer response to perceptions of hypocrisy regarding CSR, which provided insights into the research on the mechanisms of consumers’ responses to perceptions of hypocrisy regarding CSR. Previous studies have indicated that consumers’ perceptions of hypocrisy can affect their attitudes (Wagner et al., 2009) and purchase intentions (Jiang and Zhao, 2016). In the present study, consumers’ negative behaviors were introduced as a new behavioral response type. In addition to consumers’ attitudes and purchase intentions, consumers’ perceptions of hypocrisy regarding CSR also encouraged negative behaviors toward the firm. This result further enriches the knowledge regarding the types of consumer responses to perceptions of hypocrisy, leading to a more comprehensive understanding of consumers’ responses to perceptions of hypocrisy regarding CSR.

This study introduced CSR expectations, negative emotions, and negative behaviors into the study of consumers’ response mechanisms to perceptions of hypocrisy, thereby strengthening the research on the mechanism underlying consumers’ responses to perception of hypocrisy regarding CSR. The introduction of new variables and theoretical perspectives further enriches this research.

### Management Implications

Because consumers expect firms to perform CSR activities efficiently and intend to buy products from the firms implementing CSR, many firms adopt CSR strategies in order to achieve positive responses from consumers. However, improper implementation of CSR activities by many firms may lead to the formation of hypocrisy perceptions regarding CSR among consumers. Therefore, in the context of hypocrisy perception, consumers may respond negatively to CSR. The study proposes the following measures that can be taken by firms to prevent the emergence of consumers’ perceptions of hypocrisy regarding CSR and to avoid their negative impacts.

First, during CSR promotions, firms should avoid exaggerating their claims and instead be objective and realistic. Firms publicize CSR to consumers to increase consumers’ awareness regarding CSR. Consumers’ CSR expectations of the firm are generally formed during the early stages of CSR publicity. If a firm claims to conduct large-scale and high-level CSR activities, consumers are likely to form high CSR expectations from the firm. Consequently, if the firm does not implement its CSR activities as per consumers’ expectations, consumers can easily form a perception of hypocrisy regarding CSR. Therefore, firms should publicize their CSR activities appropriately so that they can achieve favorable consumers’ perceptions regarding CSR and obtain their support while avoiding the negative impact of consumers’ perceptions of hypocrisy caused by exaggerated CSR publicity.

Second, the strength of CSR activities must be consistent across firms of the same scale in the same industry. The strength of CSR activities of other firms from the same industry influences consumers’ CSR expectations of the firm. Therefore, a firm should consider the CSR performance of other firms in the same industry before implementing their own CSR activities. If other firms with the same scale in the same industry perform CSR activities effectively, consumers tend to expect good performance from the firm in question. If small-scale firms perform CSR activities favorably, consumers tend to expect large-scale firms to perform better. Consumers tend to expect the performance of the firm to be comparable to or better than that of other firms of the same scale in the same industry. Therefore, in the implementation of CSR activities, firms should intentionally observe the CSR conduct of other firms in order to maintain the same level of CSR investment with similar firms and higher level of investment than that of smaller firms. The CSR activity strength of the firm should be higher than that of other firms with the same scale in the same industry so as to avoid consumers’ perceptions of hypocrisy and thus achieve favorable outcomes.

Third, firms should improve the performance of CSR implementation and maintain effective communication with the public to increase awareness. Thus, consumers’ perceptions of hypocrisy regarding CSR can be avoided, thereby gaining their understanding and support. Therefore, in the implementation of CSR, firms should maintain sufficient investment and strive to actually solve social problems. Moreover, firms should maintain good communication with the public at all times, so that the public can be aware that the firm has undertaken effective CSR practices. Thus, firms can avoid consumers’ perceptions of hypocrisy and obtain positive responses toward the firm.

Fourth, because consumers’ negative emotions caused by perceptions of hypocrisy can directly lead to negative behaviors toward the firm, firms must strive to eliminate such emotions among consumers. The firm should first strengthen its communication with consumers, admit to its improper behaviors in the implementation of CSR, and sincerely apologize to the society. Next, the firm can further improve its CSR implementation by increasing its CSR investment. Finally, the firm can provide compensations to consumers who have suffered material and spiritual losses to further eliminate negative emotions. With effective communication, increased CSR investment, and economic compensation, consumers’ negative emotions can be eliminated to a great extent, thus reducing the possibility of consumers’ negative behaviors toward the firm.

### Research Limitations and Future Research Directions

This study discusses the psychological and behavioral mechanisms underlying the formation and effects of consumers’ perceptions of hypocrisy regarding CSR and the role of consumers’ CSR expectations and their negative emotions through a questionnaire survey. This study has the following limitations, which also indicate the future research directions. First, in the questionnaire, we adopted the method of situation simulation to describe the context and did not use the real names of corporate firms. The purpose of adopting this method was to exclude the influence of names of firms that exist in the real market. The influence of firm names on consumers’ perceptions of hypocrisy must be tested in the future. Second, the data were collected in Wuhan, China, but the response mechanisms underlying consumers’ hypocrisy perception may different in different regions. Therefore, the study conclusions must be verified in a larger geographical scope. Third, this study explores the formation mechanism of consumers’ perceptions of hypocrisy from the viewpoint of consumers’ CSR expectations. However, in addition to CSR expectation, other psychological factors may affect the formation of consumers’ perceptions of hypocrisy regarding CSR, which must be further explored in the future.

## Data Availability Statement

The raw data supporting the conclusions of this article will be made available by the authors, without undue reservation.

## Ethics Statement

Ethical review and approval was not required for the study on human participants in accordance with the local legislation and institutional requirements. Written informed consent from the participants was not required to participate in this study in accordance with the national legislation and the institutional requirements.

## Author Contributions

All authors listed have made a substantial, direct, and intellectual contribution to the work, and approved it for publication.

## Conflict of Interest

The authors declare that the research was conducted in the absence of any commercial or financial relationships that could be construed as a potential conflict of interest.

## Footnotes

1 https://cul.sohu.com/20110428/n306651374.shtml

2 http://gongyi.sina.com.cn/gyzx/2009-08-11/113711965.html
